# Genomic exaptation enables *Lasius niger* adaptation to urban environments

**DOI:** 10.1186/s12862-016-0867-x

**Published:** 2017-02-07

**Authors:** Evgenii A. Konorov, Mikhail A. Nikitin, Kirill V Mikhailov, Sergey N. Lysenkov, Mikhail Belenky, Peter L. Chang, Sergey V. Nuzhdin, Victoria A. Scobeyeva

**Affiliations:** 1 Faculty of Biology, Department of Evolution, Lomonosov Moscow State University, Moscow, Russian Federation; 2 Faculty of Bioengineering and Bioinformatics, Lomonosov Moscow State University, Moscow, Russian Federation; 3 Belozersky Institute for Physicochemical Biology, Lomonosov Moscow State University, Moscow, Russian Federation; 4 Molecular and Computational Biology, University of Southern California, Los Angeles, CA 90089 USA; 5 Department of Applied Mathematics & Mathematical Biology and Bioinformatics Laboratory, St.Petersburg State Polytechnical University, St.Petersburg, 195251 Russia

**Keywords:** *Lasius niger*, Urban environment, Draft genome, Transposable element, Detoxification, Directional selection

## Abstract

**Background:**

The world is rapidly urbanizing, and only a subset of species are able to succeed in stressful city environments. Efficient genome-enabled stress response appears to be a likely prerequisite for urban adaptation. Despite the important role ants play in the ecosytem, only the genomes of ~13 have been sequenced so far. Here, we present the draft genome assembly of the black garden ant *Lasius niger* – the most successful urban inhabitant of all ants – and we compare it with the genomes of other ant species, including the closely related *Camponotus floridanus*.

**Results:**

Sequences from 272 M Illumina reads were assembled into 41,406 contigs with total length of 245 MB, and N50 of 16,382 bp, similar to other ant genome assemblies enabling comparative genomic analysis. Remarkably, the predicted proteome of *L. niger* is significantly enriched relative to other ant genomes in terms of abundance of domains involved in nucleic acid binding, DNA repair, and nucleotidyl transferase activity, reflecting transposable element proliferation and a likely genomic response. With respect to environmental stress, we note a proliferation of various detoxification genes, including glutatione-S-transferases and those in the cytochrome P450 families. Notably, the CYP9 family is highly expanded with 19 complete and 21 nearly complete members - over twice as many compared to other ants. This family exhibits the signatures of strong directional selection, with eleven positively selected positions in ligand-binding pockets of enzymes. Gene family contraction was detected for several components of the olfactory system, accompanied by instances of both directional selection and relaxation.

**Conclusions:**

Our results suggest that the success of *L. niger* in urbanized areas may be the result of fortuitous coincidence of several factors, including the expansion of the CYP9 cytochrome family due to coevolution with parasitic fungi, the diversification of DNA repair systems as an answer to proliferation of retroelements, and the reduction of olfactory system and behavioral preadaptations from non-territorial subdominant life strategies found in natural environments. Diversification of cytochromes and DNA repair systems along with reduced odorant communication are the basis of *L. niger* pollutant resistance and polyphagy, while non-territorial and mobilization strategies allows more efficient exploitation of large but patchy food sources.

**Electronic supplementary material:**

The online version of this article (doi:10.1186/s12862-016-0867-x) contains supplementary material, which is available to authorized users.

## Background

Ants are a successful and diverse group of insects, playing a role in various ecosystem functions. These roles include facilitating soil aeration, preying on herbivore insects, farming aphids for their honeydew, and cutting leaves to farm fungi for foraging. Ant eusociality is of great scientific interest and numerous works have addressed issues in its division of labour and cast-specific gene expression. Most ant genomes were sequenced due to their status as an invasive species (*Solenopsis invicta* [1], *Linepithema humile* [2] and *Cerapachys biroi* [3]), for their unusual cast division of labour (*C. biroi* and *Harpegnatos saltator* [4]), or for their negative impact on the economy (*Camponotus floridanus* [4]).

The black garden ant Lasius niger is a usual ant of the northern hemisphere and dwells in nearly all landscapes in Europe, ranging from forests to big cities. The last review of *L. niger* was in [5], where the new species *Lasius platythorax was* established. Workers in *L. niger* are small (ca. 5 mm), while queens can reach 16 mm. Colonies are monogynous and have only one egg-producing queen, though polyandry does occurs [6, 7]. *L. niger* nests are usually underground or in sand mounds, but are also found in wall cracks and rotting wood. *L. niger* feeds primarily on honeydew and other sugar-rich substances; small invertebrates are also part of their diet. Farming aphids for honeydew often occur in gardens, thus effectively making itself a pest. These ants are also known to infest human housing, causing noticeable disturbances [7]. *L. niger* dwells in Holarctic region, inhabiting all of Europe and parts of Asia and North America. *L. niger* is the most widespread of all sequenced ants.

In natural environments *Lasius niger* occupy a subdominant position in ant communities, while species of the genera *Myrmica* and *Formica* play the main role as generalist predators. When forest ecosystems are eroded, however, dominant ant species are the first to disappear, creating possible niches and opportunities for other ant species. *Lasius niger* has a very flexible ecological strategy and can inhabit different spatial tiers from soil to trees. Due to its ecological plasticity *Lasius niger* has grown widespread in urbanised areas: the black garden ant had even increased its abundance in highly affected territories [8].

Here we report the draft genome of *L. niger* as a means of studying the genetic mechanisms underlying insect adaptation to urban environments. We focus on gene families that could facilitate behavioural or physiological means of coping with specific urban stresses.

## Methods

### Source material

Population samples for sequencing were obtained from two locations: near the campus of Moscow State University and from the Istra district ca. 70 km from Moscow. In early September 2011 worker ants from three sites (five nests from each site) at both locations were collected using aspirators and stored separately in 98% ethanol. Approximately 50–60 ants from each of six sites were treated as different samples. For exact sample sizes see Additional file 1: Table S1. Samples were dry-freezed and transported to the University of Southern California.

### Genomic procedures

Genomic DNA for each of the six samples were prepared into six libraries (one per site) and sequenced as paired-ended 100 bp reads on an Illumina HiSeq 2500. Each library was individually barcoded and sequenced as a pooled sample of barcoded DNA, generating 423 million (M) total reads.

Since *de novo* assembly of complete genomes can be sensitive to sequencing errors, it is critical that the reads used to generate contigs have the highest sequencing quality. Reads were removed from consideration in the de novo assemblyif the read had a terminal “phred” [9] (quality value less than 15), or if the read contained more than two unknown nucleotides. Reads were also filtered out with Trimmomatic [10] if they had similarity to known polymerase chain reaction primer and Illumina adapter sequences.

The 272 M retained reads from all samples were assembled with SPAdes version 3.0.0 [11]. The data are deposited to Bioproject PRJNA171386.

Genome assembly completeness was assessed using CEGMA [12] and BUSCO [13]. CEGMA score was 98,8% compared to 98,0–99,6% range for other ant genomes [3]. The raw Illumina data and the draft assembly have been deposited in NCBI under the Bioproject PRJNA171386 and will be released upon publication.

### Gene prediction and Gene Ontology (GO) content

For gene prediction AUGUSTUS [14] was used with the *Nasonia vitripennis* genome as a training set. The prediction was done on both strands, including partial genes. Predicted genes were searched against the NR protein database (BLASTP, max_target_seqs 20). Contigs with bacterial and fungal genes were removed from the assembly. Bacterial 16 s RNA were identified using BLAST searches against the bacterial 16 s RNA database; selected sequences were then searched against the NR database for genus and species identification.

For more accurate searches of genes encoding cytochromes, odorant receptors, odorant-binding proteins and desaturases, hymenopterans protein sequences were used to build alignments using hmm model with hmmbuild. We built separate models for each major CYP superfamily (CYP4, CYP6, CYP9). Hmmsearch output was checked by BLAST (nr) and delta-blast for the presence of specific domains. Genes with hmmersearch hits were aligned using BLASTP against the NR database, and genes with the majority of hits from other families were removed from further analysis. We used delta blast to check for gene-specific domains. We also searched for sequences from other examined species in the Hymenoptera Genomes Portal (for *C. floridanus*, *C. obscuritor*, *H.saltator*, *A. echinatior*, *A. cephalotes*, *S. invicta*, *P. barbatus*, *L. humile*) and in Genbank (for *C. biroi*, *M .pharaonis*, *V. emeryi*, *W. auropucntata*). Predicted proteins of *L. niger* and other ant species were then annotated using BLASTP (cutoff 5e-2) and BLAST2GO [15]. We performed GO mapping, BLAST-based annotation, Interproscan annotation and joined BLAST and Interproscan annotation data. GO terms were assigned for each protein, GO category numbers for each species were normalized by total protein number. Dixon Q-test for outliers was used to detect overrepresented and underrepresented GO groups in the *L. niger* genome, assuming it as an outlier among all ants. GO distribution was normalized with arcsine transformation [16] *L. niger* was treated as outlier in both cases - in over and under represented GO IDs. Proteins and genes of species with draft genomes that were not published yet (*C. obscuritor*, *V. emeryi*, *W. auropunctata*, *M. pharaonis*) were not included in GO-mapping and Intersproscan annotation and was used only for OR and CYP comparison and selection detection.

### Alignment and detection of selection

To eliminate potential pseudogenes, any sequence with predicted start and stop codons was discarded if it was shorter than its hypothetical product length by 25%. We assumed 300 amino acid residues for desaturases and 500 for odorant receptors and P450 cytochromes, based on rounded off mean length of respective proteins in databases. For P450 we kept only sequences containing Phe-X-X-Gly-X-Arg-X-Cys-X-Gly motif, which is believed to be crucial to stabilizing the iron ion in the heme group. The selected sequence predictions for P450, odorant receptors and desaturases were than aligned for each family using the MUSCLE software (default settings, codon alignment). For the P450 cytochrome superfamily we aligned sequences in CYP6, CYP4 and CYP9 separately for each family.

Search for best substitution models were performed in MEGA6.0. Tamura-Nei substitution modelwas found best for our dataset and was used for phylogenetic analysis. A maximum-likelihood phylogenetic tree was generated for each alignment using MEGA6.0 (gaps/missing data treatment - partial deletion, 95%,). To identify genes under selection, we applied a Z-test for selection [17] to nucleotide alignments of these genes. The positive selection hypothesis was rejected in none of the cases. A sequence was tagged as being under positive selection if both the neutrality and the purifying selection hypotheses were rejected.

For the CYP9 subfamily of P450 cytochromes we also searched for sites under selection. For this analysis we used only complete coding sequences with start and stop codons in the length range of 1425–1800 nucleotides due to limitations of selection search methods (see below). Upon removal of stop codons, sequences were codon-aligned using ClustalW software. Maximum-likelihood phylogenetic trees were then generated using MEGA6.0 (gaps/missing data treatment — complete, Tamura-Nei model, bootstrap replication number — 500, initial tree for ML — Neighboor Joining). Based on these trees, a selection search with FUBAR (*p* < 0.05) and MEME (*p* < 0.05) methods was performed for each codon in a given alignment using HyPhy2.0 software. The number of amino acid substitution events were then calculated based upon ancestor sequence reconstructed with SLAC.

## Results

### Genome assembly and annotation

We sequenced and annotated the draft genome of *Lasius niger* and compared it with the draft genomes of other ants. The 272 M reads were assembled with SPAdes [11] into 41,406 contigs of more than 500 bp in length with a total length 245 MB and an N50 of 16,382 bp. These figures are similar to other available ant draft genomes and enable comparative genomic analysis [1, 4]. The assembly also included 30,191 contigs of more than 1000 bp in length with a total length 237 MB. CG content was found to be 38%, similar to that found in *Camponotus floridanus* (34%) [4]. Median kmer coverage is 19, exhibiting a bimodal distribution of kmer coverage with peaks at 1 and 46.CpG dinucleotides are overrepresented in the *L. niger* genome: observed/expected CpG ratio is 1.55, similar to other ants (12):

We identified transposable elements throughout the L. niger genome using RepeatMasker 4.0.6 [18] against the Repbase TE library v2016-08-29 [19]. We found 3.47 MB of known transposable elements (compared to 8.6 MB in *C. floridanus* and 30 MB in *H. saltator* using same method). However, there are many intact ORFs of transposon proteins compared to other ants (see «GO distribution» below), which suggest high recent transposon activity in the *L.niger* lineage. Transposons detected by RepeatMasker include 531 LINEs, mostly from R1/LOA/Jockey family, and 2858 LTR elements (1513 Gypsy/DIRS1, 755 BEL/Pao and 590 Ty1/Copia).

Application of the gene prediction tool Augustus [14] revealed 19,989 genes in *L. niger* contigs, with 15,350 having both start and stop codons. Annotated genes were shorter than in other ant species and contained fewer exons, but total exon number and length is similar to values observed in other ants (Additional file 1: Table S13). Therefore we conclude that the gene space of *L. niger* annotation is as complete as other ants, although the genome is more fragmented.

To distinguish between genes in the ant genome and those originating from microbes, we used blast search against the non-redundant protein database. Analysis of 16S and 18S ribosomal RNA genes revealed the following non-ant species: 8 bacteria (*Serratia marcescens, Luteibacter sp., Gluconobacter sp., Spiroplasma sp, Acetobacter sicerae, Georgenia sp., Tsukamurella paurometabola*), one enthomopathogenic fungus *Ophiocordyceps unilateralis*, an unidentified spider and a plant from family *Fabiaceae*. High protein blast-hit similarity additionally identified Enterobacteriacea (*Serratia* + *Yersinia*/*Klebsiella*), *Propionobacter*, nematode, *Lactobacillus*, actinomycete (probable Strepomyces), and, potentially, *Fusarium* (see Additional file 1: Table S2). After removing hits to bacterial and fungal contigs we obtained a total of 18,247 ant genes; 12,207 potentially complete and 6040 partial sequences. All these contigs were also removed from the BioProject.

**Fig. 1:**
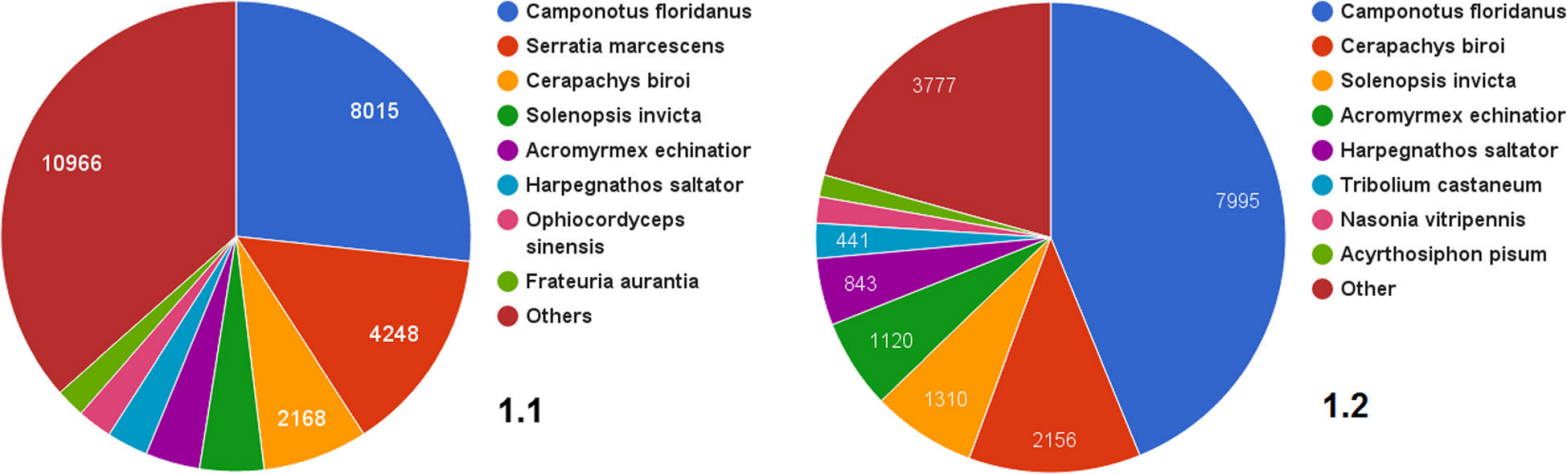
Top blast hits of *L. niger* metagenome (1.1) and genome (1.2) assembly. Most hits are from *C. floridanus*, closest sequenced relative of *L. niger*, and other ants

Among non-bacterial annotated contigs the vast majority (37%) are annotated as proteins, common with *Camponotus floridanus* (Fig. 1). *C. floridanus* belongs to the same family Formicinae as *L. niger* and their similarity in proteins corresponds to morphological likeness. Three other ant species from the Fig. 1 – *Solenopsis invicta*, *Acromyrmex echinator* and *Harpegnatos saltator* are highly specialized and diverged from *Formicinae* lineage in late Cretaceous [20].

### GO distribution

The predicted proteome of *L. niger* is significantly (*p* < 0,05) different from other ant genomes in terms of abundance of several GO categories. The proteome is enriched in domains involved in nucleic acid binding (GO:0003676), DNA repair (GO:0006281), nucleotidyl transferase activity (GO:0016779), heterocyclic compound binding (GO:1901363) and contain fewer domains involved in neuropeptide signaling (GO:0007218). *L. niger* might also contain fewer odorant binding domains than other ants (77 versus 114–264), but this difference was not statistically significant in a Q-test.

Overrepresentation of nucleic acid binding domains is due to high abundance of retrotransposon genes coding gag-pol polyproteins or separate reverse transcriptases. We detected 475 pol and gag-pol genes in the *L. niger* genome compared to 55 in Camponotus and between 5 and 42 in other ant species. No env genes were found to match the abundance of pol and gag-pol. We conclude that the pol and gag-pol genes belong to retrotransposomes and are not from ongoing retroviral infections.

DNA repair domains are overrepresented in *L. niger* mostly because of duplications of PIF1-like helicases. The *L. niger* genome encodes 15 PIF1-like helicases compared to 3 in *Camponotus* and 1 in most other ants. Other DNA repair proteins duplicated in *L. niger* include chromosome structure maintenance proteins 1A and 2, DNA damage-binding protein 1 and tyrosyl-phosphodiesterase.

### Detoxification system genes

As our focus is on potential genomic features enabling success in urban environments, we paid special attention to the gene families involved in detoxification. Glutathione-S-transferases and Cytochrome P450 families are the most common detoxification genes among hymenoptera [21] and we examined these two families in detail. We expected to find multiple duplications in these families, and in glutathione-S-transferase family we found 15 genes, (Additional file 1: Table S3), though most had incomplete and short sequences. We found no evidence of species-specific duplications of glutathione-S-transferase in the *L. niger* genome.

We did find CYP genes being highly duplicated in *L. niger*, with 72 sequences matching CYP genes across 11 families (only complete and mostly complete genes with >250aa CDS were analyzed). This is the second largest value among ants, second only to 76 CYP450 genes in *C. floridanus* from the same family (Additional file 1: Table S4). Compared to *A. mellifera* we see fewer CYP families, but more genes per family in those present (only 40 CYP450 genes found in *Apis*). Interestingly, there are 15 tandem clusters of CYP450 genes found in the *L. niger* genome assembly, most of which are members of CYP9E subfamily.

**Fig. 2:**
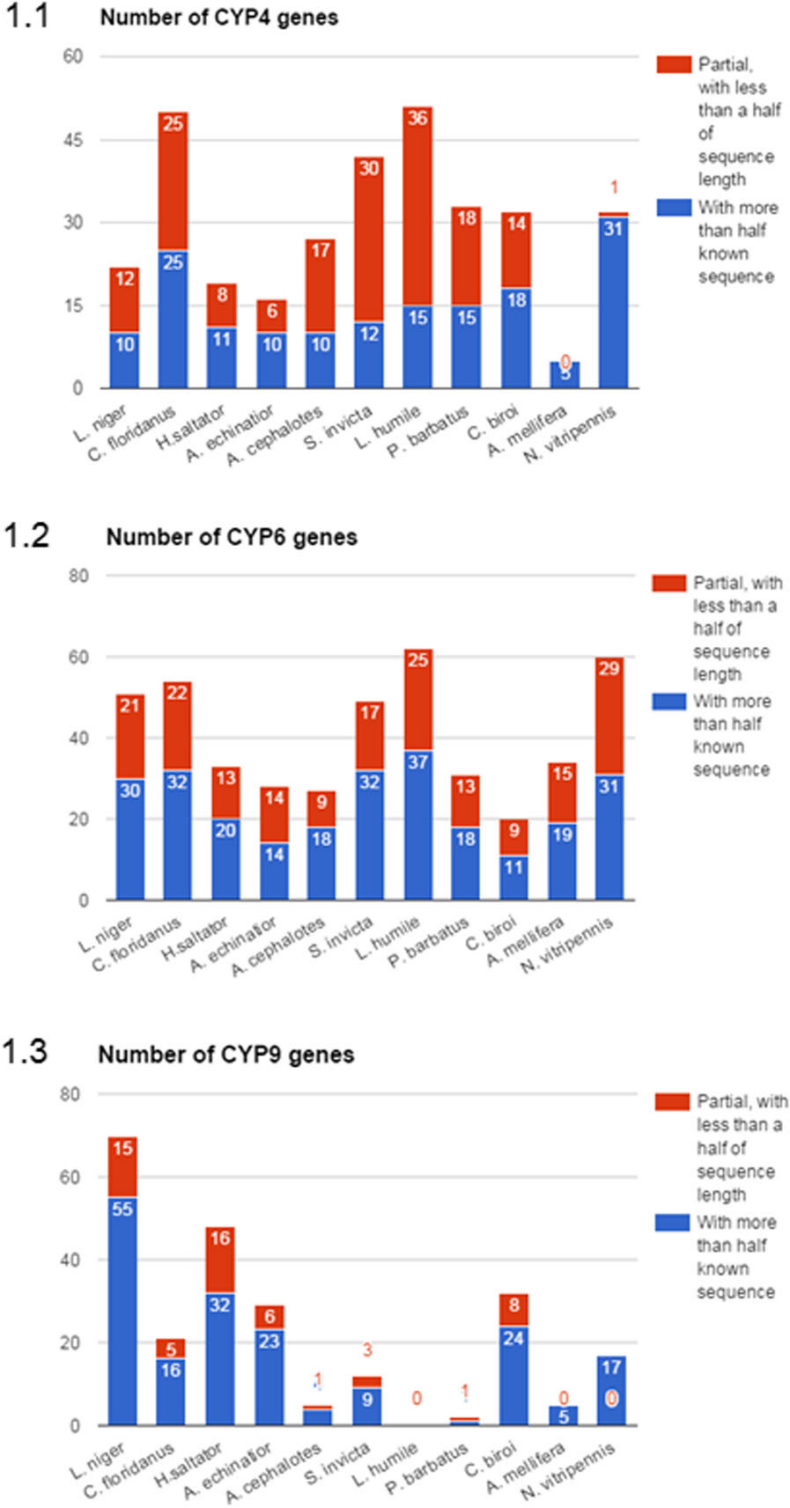
Comparative abundance of CYP450 subfamilies in hymenopteran genomes. Various subfamilies are expanded in different species. Even closely related *C. floridanus* and *L. niger* differ significantly in relative abundance of CYP6 and CYP9 subfamilies

Most CYP450 genes in ant genomes are members of cytochrome P450 9th, 6th and 4th families (Fig. 2). The CYP9 family is highly expanded in L. niger, with 19 complete and 21 nearly complete members - over twice as much as any other ant. The number of CYP4 and CYP6 gene families in L. niger genome is within the diversity range of other ants. Genes of the 9e2 family are organized in 12 duplicated clusters, with genes of 6 K, 6A and 4G families also exhibiting tandem duplications.

### Positive selection in cytochrome P450 genes

For phylogenetic analyses and selection tests we selected only complete and nearly complete (>250 codons) P450 genes where the conservative heme-binding motif Phe-X-X-Gly-X-Arg-X-Cys-X-Gly was present (29 out of 40 CYP9, 5 out of 9 CYP6, and 8 out of 10 CYP4 for *L. niger*).

**Fig. 3:**
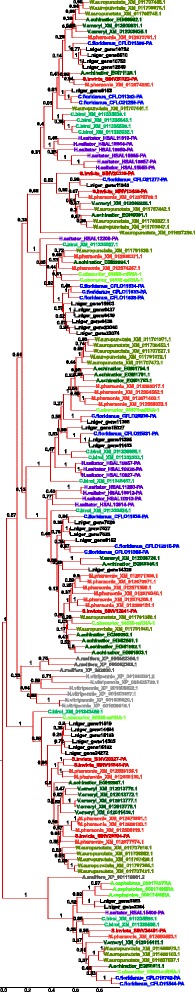
Phylogenetic tree of hymenopteran CYP9 genes. Branches where statistically significant positive selection was found by Z-test are in *red*. Positive selection was found in most of the terminal branches of the phylogenetic tree. Many lineage-specific duplications are evident in *L. niger, A. echinator* and *C. biroi*

**Fig. 4:**
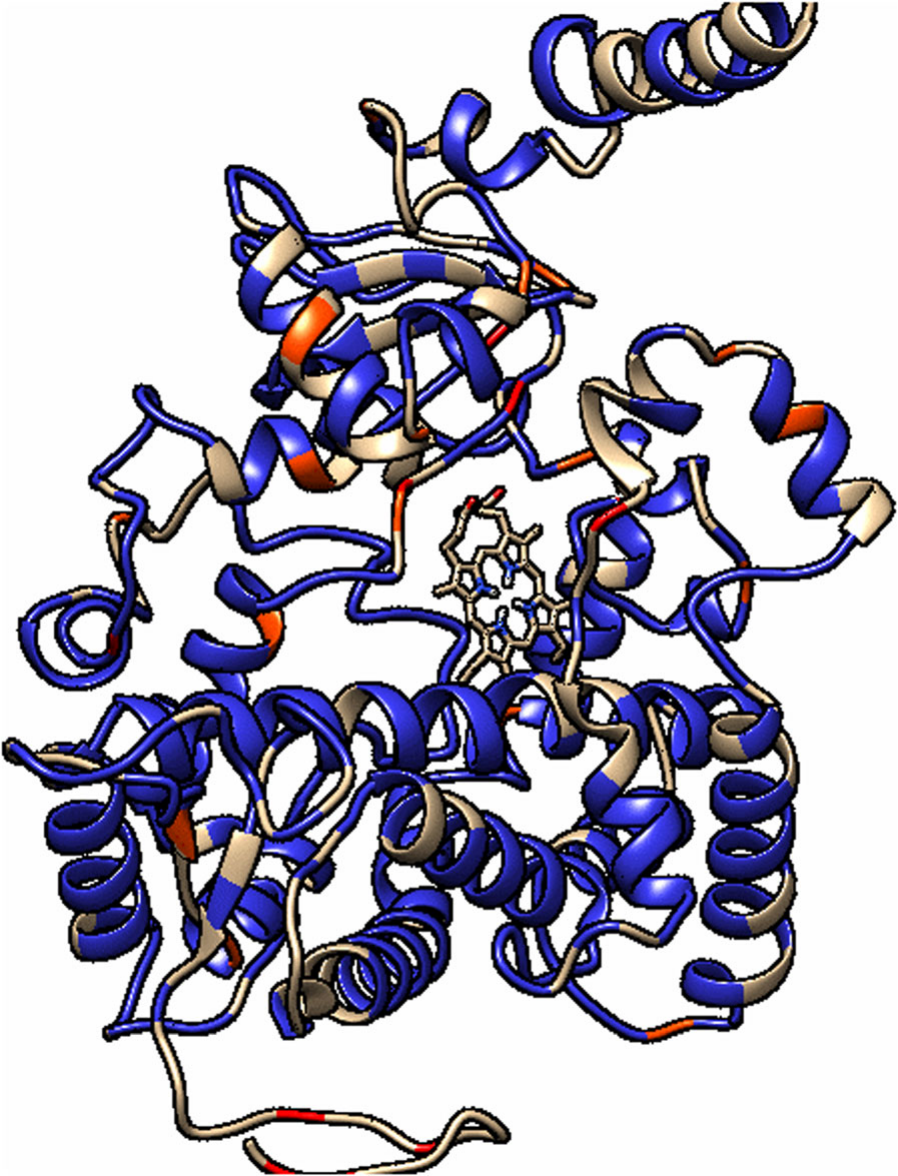
Positive (highlighted in red and orange) and negative (highlighted in blue) selected amino acids of CYP9 *L. niger.* Ligand-binding pocket is located in the upper right part of the protein molecule, with heme group in the bottom of the pocket. Most sites under positive selection are in the ligand-binding pocket, while structural alpha spiral domains are under purifing selection

In the phylogenetic tree, most *L. niger* genes form 10 clusters near those of C. floridanus, another ant in Formicinae subfamily (Fig. 3). Most of the CYP9 genes in the examined species are under positive selection according to pairwise Z test (Figs. 3 and 4, Additional file 1: Table S4). Except for recently duplicated genes phylogenetic trees of CYP4 and CYP6 always show *A. mellifera* and *N. vitripennis* as outgroups. *L. niger* contain two duplications of CYP4 genes after the divergence of *Lasius* and *Camponotus* lineages. Five *L. niger* genes of CYP6 family sit on the phylogenetic tree separately, but form clusters with *C. floridanus* genes. Two are putative pseudogenes. Most ant genes from the CYP4 and CYP6 families were under positive selection since their divergence with other hymenoptera according to a pairwise Z-test for selection

**Fig. 5:**
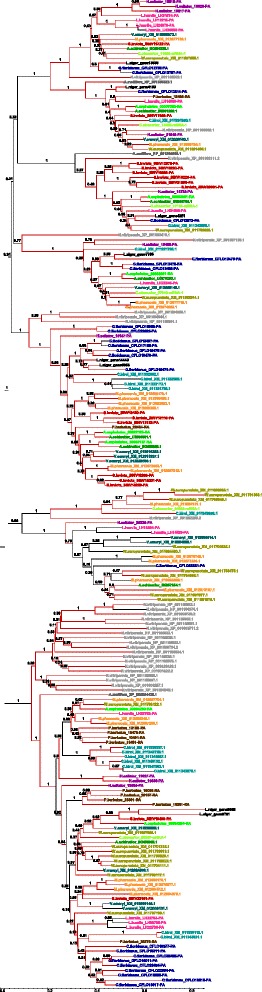
Phylogenetic tree of hymenopteran CYP4 genes. Branches where statistically significant positive selection was found by Z-test are in *red*. Positive selection was found in most of the terminal branches of the phylogenetic tree. Many lineage-specific duplications are evident in *N. vitripennis, C. floridanus* and *C. biroi*

**Fig. 6:**
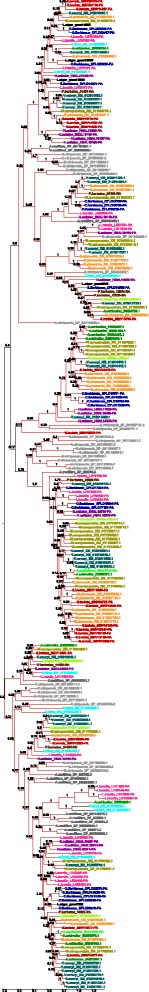
Phylogenetic tree of hymenopteran CYP6 genes. Branches where statistically significant positive selection was found by Z-test are in *red*. Positive selection was found in most of the terminal branches of the phylogenetic tree. Many lineage-specific duplications are evident in *N. vitripennis* and *A. mellifera*

As noted previously, the CYP4 and CYP6 gene families are not amplified in *Lasius niger*. Nearly all ants with sequenced genomes have between 10 and 15 fragments of CYP4 genes, coding for more than half of the protein (including between 2 and 7 full-length genes). *Camponotus* has 13 full-length genes and 12 partial genes, with the partial genes coding for more than half of the protein. Non-ant hymenopteran species *A.mellifera* and *N.vitripennis* have 5 and 32 predicted CYP4 family proteins. Despite the 30 found fragments of genes, similar to the number of CYP6 found in other species, only 9 code for more than half of the protein and only 5 have the sequence of the gem-iron stabilizing motif. Other examined species have between 12 and 133 fragments of CYP6 genes, with only 6 to 32 containing coding sequences of more than 750 bp. Every ant species except for C. obscurior has lineage specific duplication of CYP4 genes (Fig. 5). The most abundant CYP6 genes (more than 20 genes with more than half length coding region) are in invasive species (*M. pharaonis*, *V. emeryi*, *L. humile*, *W. auropunctata*, *S. invicta*), but in half of the genes the sequence of gem stabilizing motif is absent. The poorest representation is in *C. biroi* – (11 fragments total, Additional file 1: Table S4). Overall, the CYP6 tree has many species-specific duplications for all ants, except *L. niger* and *A. cephalotes* and *C. obscurior* (Fig. 6). The most amplified are CYP6 in *C. floridanus* and *S. invicta*, both having 4 groups of 3–4 genes and 4 separately lying genes. Positive selection in CYP6 family, as well as in the abovementioned families, was not significant for recent duplications; most of them are in *C. floridanus*.

### Sites under selection in CYP9

To test whether an expansion of the CYP9 family was concordant with strong evolution on gene sequences, we executed a more detailed analysis. An alignment of full-length CYP9 coding sequences between 1425 and 1800 bp with start and stop codons was used to search for sites under positive selection. These criteria identified 3 coding CYP9 sequences in *A. cephalotes*, 6 sequences in *A. echinator*, 4 in *A. mellifera*, 2 in *C. biroi*, 11 in *C. floridanus*, 13 in *H. saltator*, 19 in *L. niger*, 15 in *N. vitripennis*, 8 in *M.pharaonis*, 5 in *C.obscurior*, 1 in *V. emeryi*, 3 in *W.auropunctata* and 5 in *S. invicta*.

MEME detected 59 alignment positions under positive selection (*p* < 0,05) (Additional file 1: Table S5). Three positions were confirmed by other selection search methods (SLAC and FEL, *p* < 0.05, FUBAR with aposteriori probability >0,95), one was significant in all four methods. Negative selection was detected in 469 sites with statistical significance for FUBAR with aposteriori probability > 0,95). Eleven positively selected positions are in ligand-binding pockets of the enzyme (Fig. 4). An additional four are at the C-terminus, but this part of alignment was not present in all sequences. Most of negatively selected sites lie in structural domains of the enzyme.

The most frequent non-synonymous substitutions in CYP9 family did not change the charge and polarity of amino-acid residues (Val to Ile, Asp to Glu, Ile to Leu, Lys to Arg, Ser to Thr, Val to Ala) (Additional file 1: Table S6). Nevertheless, some substitutions changed charge and polarity – Lys to Glu, Asn to Asp, Ala to Thr and Leu and Ile to Met. The rarest were changes of Cys and Trp.

The MEME analysis found different preferred substitutions in different subtrees of the *L.niger* CYP9e tree. In one subtree, Arg was changed to Hys and Asn, while another Arg was changed to Ala. This trending of changing Arg to other amino-acids occurred only in *L. niger* alignments – in other ants not only Arg was changed, but also Ala and Hys. FUBAR analysis detects negative selection in most positions, where MEME detects positive. These sites are conservative in the majority of lineages but in other leading to *L. niger* diversifying selection did occur.

### Olfactory receptors and odorant-binding proteins

Olfaction related genes frequently exhibited signatures of directional selection or selection relaxation. Hhmsearch and reciprocal BLASTP of *L. niger* predicted proteins revealed 75 putative odorant receptors and 3 odorant-binding proteins. One putative odorant-binding protein, having high similarity with other ant OBP, is 74 amino acids in length and is not recognized by BLASTP in the REFSEQ protein database. Other ants have additional OBPs, from 7 in *C. floridanus* and *A. echinator* to 20 in *V. emeryi*. Only 31 of 74 *L. niger* predicted OR genes had coding sequence longer than 750 bp and were used for phylogenetic analysis and selection detection. Other ants have between 118 and 474 fragments of odorant receptor genes, from 49 to 335 of this manifold were used for the phylogenetic analysis. *Apis mellifera* has 127 fragments of genes and 100 fragments were used to construct the phylogenetic tree. (Additional file 1: Table S7).

Genes of odorant receptors in *L. niger* have 5 amplified clusters – 3 duplications and 2 5-genes repeats. Most tandem duplications have related sequences in other ants. We did not find any *L. niger* duplication that was species-specific (Additional file 1: Table S8). Not only did L. niger have only 26 genes of suitable quality for odorant receptors, more than half were putative pseudogenes. Despite the poor repertoire of *L. niger* odorant receptors, half were under positive selection. In contrast to the CYP genes, odorant receptors of *L. niger* do not predominantly form clusters with *C. floridanus* genes, but often cluster with genes of more distant ant species. The phylogenetic tree of odorant receptors indicates that there are many amplifications and deletions of odorant receptor genes, providing an explantation as to why closely related A. cephalotes and *A. echinator* have very different odorant receptor genes, perhaps due to pseudogenisation and lineage sorting in their putative ancestor’s gene set.

Z-test found positive selection in average in all sequence pairs.

### Fatty acid desaturases

Cuticular hydrocarbons are important molecules in ant chemocommunication. These compounds are produced by fatty acid desaturases enzymes. Ants with sequenced genomes have from 6 in *C.biroi* to 14 in *H. saltator. L. niger* and *C. floridanus* both have 11 fatty acid desaturase genes, incomplete genes not included (Additional file 1: Table S9). Desaturases of Hymenoptera are grouped into six subfamilies, with one being unique for this order and other subfamilies orthologous to five *Drosophila melanogaster* desaturases (desat1, CG9743, CG9747, CG8630 and CG15331, Additional file 2).

No orthologs of *D. melanogaster* CG15331 were found in *L. niger* and *P. barbatus*; other ants have one. Hymenopteran specific desaturases are absent from *C. biroi*. Other subfamilies of desaturases are present in all ant genomes. In all ant species, orthologs to *D.melanogaster* CG9743 are under positive selection, while there was no selection within *C. floridanus* duplications. Almost all orthologs to *D. melanogaster* CG8630 and *desat1* are positively selected as well. In hymenopteran specific desaturase group, the selection was significant predominantly in *Myrmicinae* and *Formicinae* subfamilies. No significant differences between *L. niger* and other ants can be found in desaturase gene content and selection mode (Additional file 1: Table S10).

### Telomerases

Ants are known for their great intraspecific differences in life spans and *L. niger* has the greatest: from several months in males to decades in queens [22]. In contrast to *Drosophila* in which telomere length is maintained through retrotransposition [23] and is consistent with other studied animals species, ants’ life span is based on telomerase activity. Jemielty et al. [24] showed caste-specific differences in expression level of telomerases. Our annotation contains several genes related to telomerase activity. One copy of telomerase reverse transcriptase was found. Thirteen copies of another important component of telomerase ribonucleoprotein (RNP) complex – telomerase binding protein Est1a [25] – were found (this protein was also shown to play a role in nonsense-mediated mRNA decay [26]). Four genes similar to telomerase Cajal body protein were found. Future functional analysis of telomerase sequences in *L. niger* could shed light on life span regulation in this species.

### Vitellogenins

Vitellogenins were duplicated in many formicoid lineages. Caste-specific expression is characteristic for vitellogenin paralogs in ants. However, the *C. floridanus* genome contains only one vitellogenin gene. We found only one vitellogenin in the *Lasius* genome, with high similarity to the *Camponotus* vitellogenin. HMM search for hymenopteran vitellogenins also revealed only one vitellogenin in *Harpegnatos*, *Cerapachys* and *Pogonomyrmex*. Other ant species had more vitellogenins; we found 3 vitellogenins in *Solenopsis*, 4 in *Linepithema* and *Wasmania* and 5 vitellogenins in *Volenhovia* and *Monomorium*. One more sole vitellogenin in Formicinae can not, of course, reject a hypothesis of all-Formicidae initial vitellogenin duplication, but add some more doubts to it. All mentioned vitellogenins were vgg, we did not search for vgf.

## Discussion

### High loads of fungal parasites and retroelements

Here, we assembled the draft genome of *L. niger* and compared it with the genomes of other ant species. Surprisingly, retrotransposons are an order of magnitude more abundant in this ant compared to others. Discovered retrotransposon genes do not contain internal stop codons and expected to be active. We also discovered the expansion of DNA repair proteins. Here, we hypothesize that expansion of DNA repair proteins could be a genome response to the expansion of retroelements. DNA repair system takes part in transposon integration and is required to restore DNA damage caused by incorrect integration. TEs are known to interfere with host repair, therefore the diversification of DNA repair system may represent the host’s response to retrotransposon expansion [27]. We further hypothesize that the diversification of DNA repair system might be an exaptation pre-adapting *L. niger* to explore urban environments. Specifically, enhanced DNA repair enzyme repertoire may play a role in resistance against DNA-damaging xenobiotics such as polycyclic aromatic hydrocarbons.

Furthermore, both protein and 18S rRNA BLAST results show high level of fungal DNA from genus *Ophiocordyceps*. These fungi are well-known entomopathogens [28], infecting various insects including ants. Fungal 18 s rDNA from *L. niger* assembly is most similar (95%) to sequences from *O. unilateralis* infecting *Camponotus* carpenter ants, suggesting high rate of fungal infection in *L. niger*. However, characteristic fruit bodies protruding from the dead ant’s head have never been seen in *L. niger*. It was shown that laboratory settings that *Ophiocordyceps* infects and kills many ant species besides its natural host [29] However, reproduction and spore dispersal of *Ophiocordyceps* requires complex host manipulation (infected ants attach to tree twigs in death grip), which is successful only in its natural host. Two species of *Camponotus* (*C. herculeanus* and *C. fallax*) live in the Moscow region [30] and they may be natural hosts of *Ophiocordyceps* found in *L. niger*. Alternatively, *L. niger* might be a natural host for this fungus but its transmission does not involve typical fruit body formation. Nevertheless, the observed fungal DNA level suggests that *Ophiocordyceps* infection may be an important cause of mortality among *L. niger* and there may be the selective pressure toward resistance to this pathogen.

### CYP9 expansion

Insects can cope with pollution in two ways - physiological or behavioral. Metabolic detoxification of xenobiotics is an example of physiological adaptation. We studied the cytochrome P450 superfamily in detail, as it is known for its crucial role in resistance to xenobiotics [31, 32]. Compared to other ants, *L. niger* has a highly amplified CYP9 subfamily. Phylogenetic analysis and comparison of CYP9 genes of *L. niger* with *A. mellifera* and *N. vitripennis* allocated all genes of this subfamily in ants into subfamilies CYP9E and CYP9P. Functions of these subfamilies are unknown, but closely related subfamilies (CYP9F and CYP9G) are known for their roles in insecticide resistance [33, 34].

Entomopathogenic *Cordycepitales* are known to synthesize wide range of toxic compounds [35, 36]. We have previously shown using molecular docking and virtual screening [37] that *L. niger* CYP9 cytochromes form complexes with low free energy with fusarin C, fumonisin, ergot-alkaloids, enniatin and beauvericin. These compounds are produced by *Ophiocordyceps* and are important pathogenicity factors [28, 36, 38]. For some of *L. niger* CYP9 free energy of complexes with fusarin are below −480 kJ/mol that suggests high affinity of enzymes to this substrate. We hypothesize, therefore, that the expansion of CYP9 in *L. niger* genome may result from coevolution with enthomopathogenic fungi.

Cytochrome P450 superfamily evolution in Formicidae was extensively studied [39]. Differential expansions and contractions of P450 subfamilies were observed in different genera. These changes are linked with adaptations to novel ecological niches and changes in colony lifestyle. Trophic specialization was found to promote the contraction of P450 family in *P. barbatus* [40].

*L. niger* and *C. floridanus*, which belong to the subfamily *Formicinae*, contain approximately the same number of cytochrome P450 genes, and this number is higher than that in other species of ants. Like *L. niger*, *C. floridanus* dwells in an urban environment and similarly omnivorous [41]. Its diet includes living and dead insects and the secretions of aphids and scale insects [42]. Cytochrome P450 genes have wide range of substrates and single amino acid substitution can drastically change substrate specificity [43]. It is possible that the large set of cytochrome P450 genes provide for polyphagia by enabling specific and nonspecific neutralization of various phyto and mycotoxins (phytotoxin resistance would enable *L. niger* to feed on nectar and resistance to mycotoxins would enable it to feed on dead insects). Cytochrome P450 genes may also be important in resistance to urban pollutants of different chemical composition (polycyclic hydrocarbons, plastics, and petrochemicals). Some cytochrome P450 genes can oxidize some of these chemicals, e.g., benzo(a)pyren [44].

The importance of detoxification system in *L. niger* xenobiotic resistance may be further increased due to scarcity of anti-fungal protective behaviour. For example, *L. niger* does not remove aphids infected with parasitic fungi from symbiotic aphid colonies, while this behaviour is common in other aphid-herding ants such as *Formica s. str*. (Novgorodova, in press).

### Odorant communication

Genes of odorant receptors and odorant-binding proteins, including partial sequences, appear to be less abundant in the assembly of *L. niger* than in other ant genomes. We hypothesize that the *Lasius* genus lost some odorant receptor and odorant-binding genes in the course of their evolution.

Our reanalysis of previously published data enables unbiased comparison between species. In *S. invicta,* there are only 151 fragments of odorant receptors and odorant-binding proteins, instead of 297 complete genes and more than 100 fragments that were previously reported [1]. This observation could be explained by the fact that the insect odorant receptors were considered to be homologous to odorant receptors of vertebrates prior to 2013 [45]. Accordingly, G-protein coupled receptors were treated as hypothetically odor detection related, while now we know that all insect odorant receptors are ion channels. Another published prediction that was not confirmed in our work, is an increase in amounts of odorant receptors in ants as compared to *A. mellifera* and *N. vitripennis*. On the contrary, we observed that, in ants, odorant receptors and odorant-binding protein families underwent gene loss. This loss may be caused by transition of ants to terrestrial lifestyle and, therefore, increase in the role of antennal contacts in species communication (J. Reznikova, pers. Comm.). *L. niger* was shown to prefer visual information over pheromone trails when these stimuli were in conflict, contrary to other ant species [46, 47]. Specifically in respect to urban environments, odorant receptor loss can be very useful, as it is increasing the resistance of the species to repellents.

## Conclusions

In conclusion, we hypothesize that a number of seemingly unrelated events – expansion of DNA repair, olfactory system genes loss and CYP9 gene amplification - can be viewed as preadaptations required for the successful colonization of urban areas. Among these preadaptations, the changes in olfactory system genes and CYP genes provide two different ways to fight against xenobiotics. The latter is achieved rather by avoiding poisonous chemicals versus metabolizing them with the help of detoxication system, thus, opening the potential for trade-off between these two systems. Moreover, this hypothesis is experimentally supported [48] by a negative correlation between CYP and odorant-binding proteins expression levels in insects. In our future work, we will test whether urban-life associated selection sweeps are enriched in the genes that comprise xenobiotic avoidance and metabolizing systems.

## Additional files

Additional file 1: Tables S1-13. Samples, number of CYP, OR, OBP and desturases genes, results of selection detection, neuropeptides. (XLSX 47 kb)
Additional file 2: Phylogenetic tree of ants fatty acid desaturases. 1 - hymenoptera-specific desaturases, 2 D. melanogaster CG9747 orthologs, 3 — D. melanogaster CG9743 orthologs, 4 — D. melanogaster desat1 orthologs, 5 — D. melanogaster CG8630 orthologs, 6 — D. melanogaster CG15331 orthologs. (PDF 2930 kb)
