# Supplementary figures and images for: Genomic exaptation enables Lasius niger adaptation to urban environments
Source: BMC Evol Biol. 2017 Feb 7;17(Suppl 1):39. doi: 10.1186/s12862-016-0867-x (PMC5333191; doi:10.1186/s12862-016-0867-x)

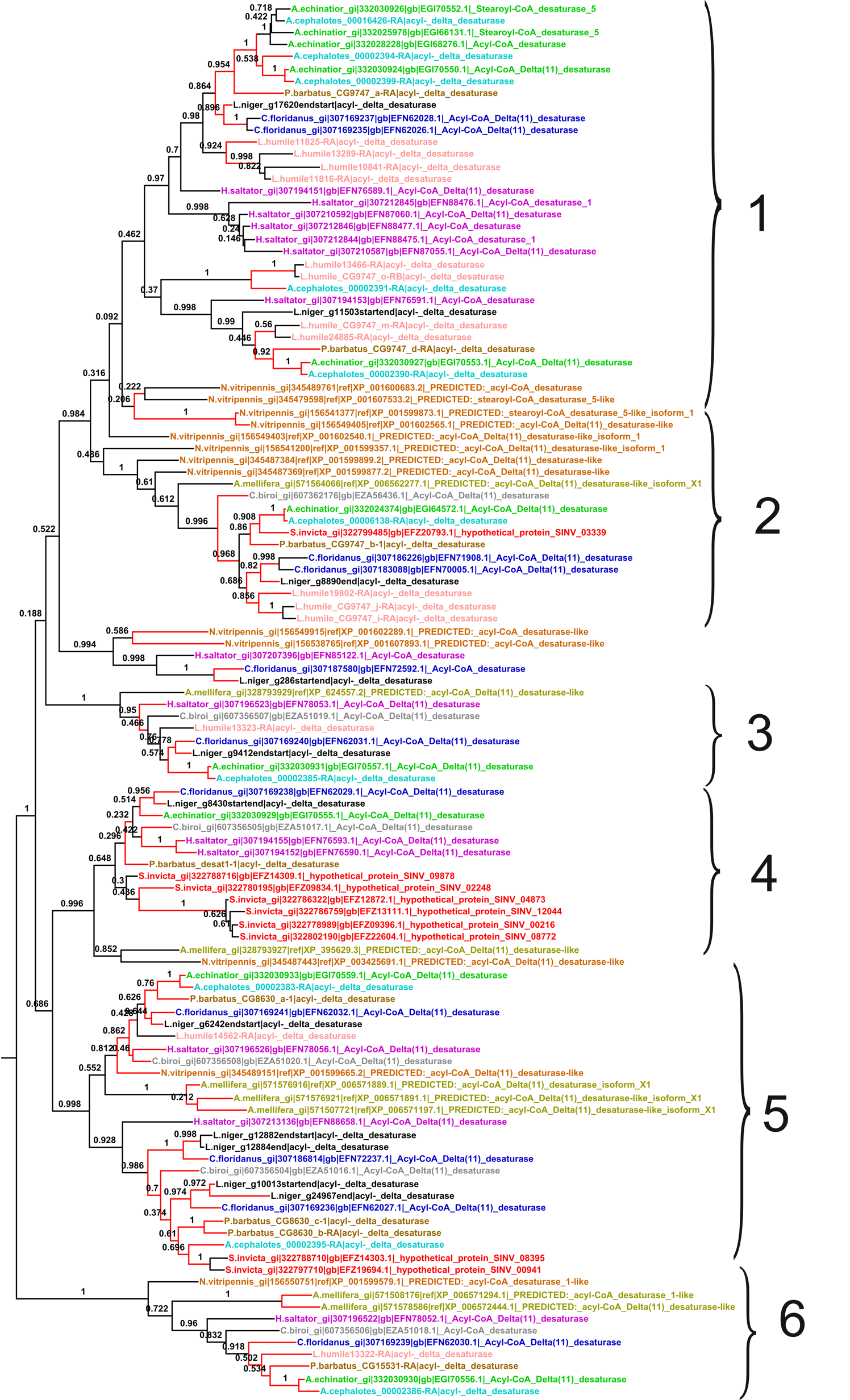

Supplement: Additional file 2: — Phylogenetic tree of ants fatty acid desaturases. 1 - hymenoptera-specific desaturases, 2 D. melanogaster CG9747 orthologs, 3 — D. melanogaster CG9743 orthologs, 4 — D. melanogaster desat1 orthologs, 5 — D. melanogaster CG8630 orthologs, 6 — D. melanogaster CG15331 orthologs. (PDF 2930 kb) [file 12862_2016_867_MOESM2_ESM.pdf]
